# Muscles Affecting Minimum Toe Clearance

**DOI:** 10.3389/fpubh.2021.612064

**Published:** 2021-05-31

**Authors:** Chamalka Kenneth Perera, Alpha Agape Gopalai, Siti Anom Ahmad, Darwin Gouwanda

**Affiliations:** ^1^School of Engineering, Monash University, Selangor, Malaysia; ^2^Malaysian Research Institute on Ageing, Universiti Putra Malaysia, Selangor, Malaysia

**Keywords:** minimum toe clearance, tibialis anterior, gastrocnemius, surface electromyography, aging, gait, joint angle

## Abstract

The aim of this study was to investigate how the anterior and posterior muscles in the shank (*Tibialis Anterior, Gastrocnemius Lateralis and Medialis*), influence the level of minimum toe clearance (MTC). With aging, MTC deteriorates thus, greatly increasing the probability of falling or tripping. This could result in injury or even death. For this study, muscle activity retention taping (MART) was used on young adults, which is an accepted method of simulating a poor MTC—found in elderly gait. The subject's muscle activation was measured using surface electromyography (SEMG), and the kinematic parameters (MTC, knee and ankle joint angles) were measured using an optical motion capture system. Our results indicate that MART produces significant reductions in MTC (*P* < α), knee flexion (*P* < α) and ankle dorsiflexion (*P* < α), as expected. However, the muscle activity increased significantly, contrary to the expected result (elderly individuals should have lower muscle activity). This was due to the subject's muscle conditions (healthy and strong), hence the muscles worked harder to counteract the external restriction. Yet, the significant change in muscle activity (due to MART) proves that the shank muscles do play an important role in determining the level of MTC. The *Tibialis Anterior* had the highest overall muscle activation, making it the primary muscle active during the swing phase. With aging, the shank muscles (specifically the *Tibialis Anterior*) would weaken and stiffen, coupled with a reduced joint range of motion. Thus, ankle-drop would increase—leading to a reduction in MTC.

## Introduction

With an increasing elderly population worldwide, the number of fall and tripping related injuries have also increased (1). This poses a serious threat to the health and standard of living of the elderly. The occurrence of such accidents can also, negatively impact the economy—where society must bear the cost (1, 2). Due to this, it is vital that the underlying causes for these accidents are understood, so that precautionary measures or devices can be implemented.

There is sufficient evidence to show that one of the main causes for tripping and falling, is a low minimum toe clearance (MTC) (3, 4). This gait event is defined as being the smallest vertical distance between the ground and the lowest point of the foot (toes), during the swing phase. The swing phase, occurs between a toe-off (TO) and a heel-strike (HS) (5).

Multiple causes for a low MTC, both in adults and the elderly have been investigated through various studies. One such study, showed that dual task walking and low cognitive awareness were factors linked to a reduction in MTC (6). On the other hand, Mills et al. (4) showed that a lower step length and a slower walking velocity led to a decrease in MTC.

It is established that the primary ankle extensors and flexors were responsible for disorders such as drop foot, which led to a lower toe clearance (TC) (7). The anterior and posterior muscle groups associated with the ankle's movements are the *Tibialis Anterior* (responsible for dorsiflexion) and the *Gastrocnemius* (responsible for plantar flexion) in the shank. The *Gastrocnemius* is split into two subdivisions—the Lateralis and Medialis. With aging, a person's muscles would weaken and stiffen, along with a reduction in the range of motion (ROM) and flexion of their joints (8, 9). This results in a weaker ankle with greater foot drop, possibly leading to a reduction in MTC.

It was hypothesized, that the *Tibialis Anterior* would be the prominent muscle active (in the shank) during the swing phase, as compared to the *Gastrocnemius*. Thus, would play a significant role in influencing the level of MTC.

Furthermore, it was predicted that, with aging, the MTC would decrease, and this would be a result of weaker anterior and posterior shank muscles (lower muscle activity).

Young adults participated in this study using muscle activity retention taping (MART) techniques; to simulate aging gait on a muscular level (5). Hence, elderly subjects were not required as similar gait characteristics could be replicated. The validity and reliability of MART was discussed in reference (5). The hypothesis of this study differs from that in (5), as it aims to investigated the extent to which the shank muscles affect MTC, along with joint kinematics. The report herein, details the methodology, results, and conclusions from the conducted study.

## Materials and Methods

### Subject Details

**Figure 1:**
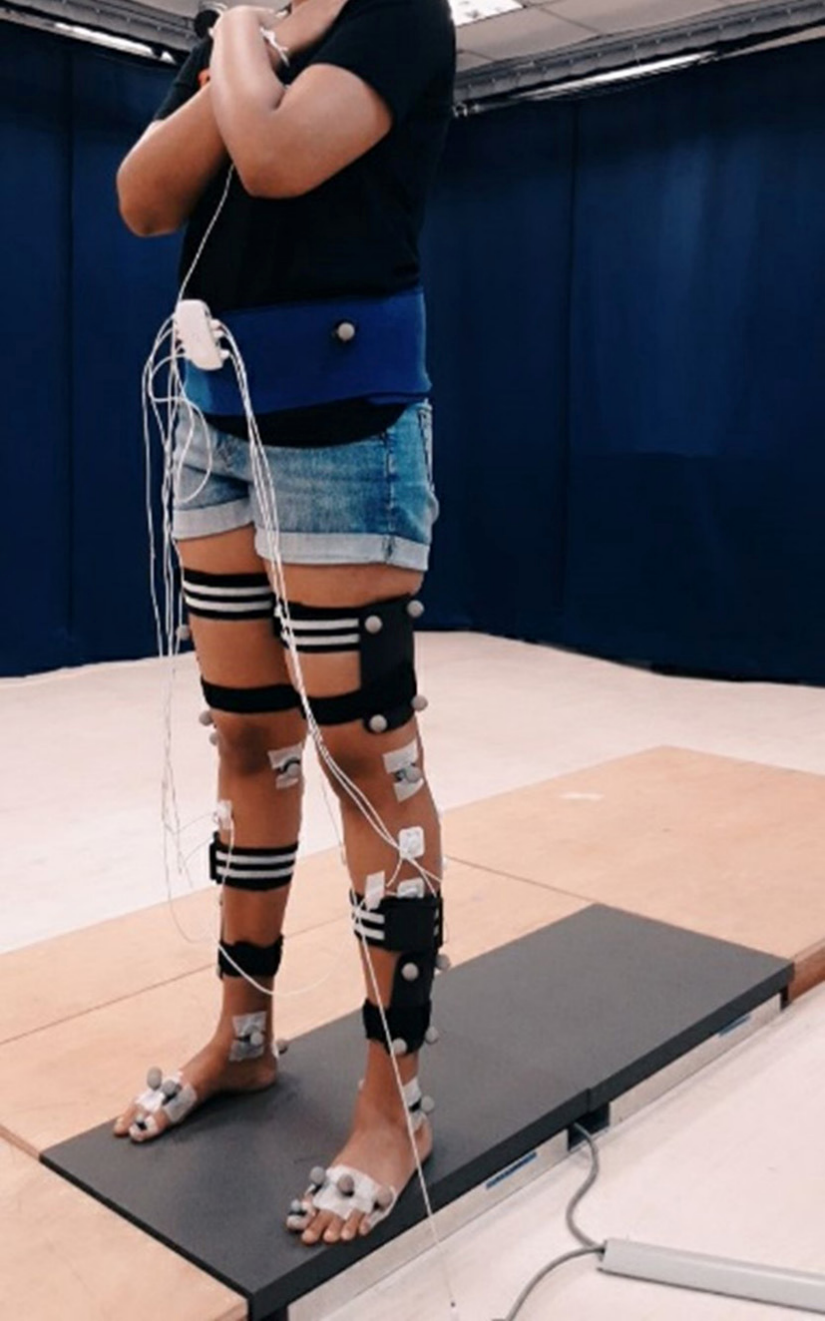
Static trial for a subject with the placement of motion capture markers, Biosignalsplux SEMG electrodes and MART bands.

Data from five subjects were considered. Their average age, weight and height were 22.60 ± 1.52 years, 75.4 ± 11.30 kg and 1.70 ± 0.034 m, respectively. Ethical approval was obtained from the Monash University Human Research Ethics Committee (Project number: 23200). Each subject provided informed consent, prior to the trials, and were recruited *via* word of mouth (within the student community) from Monash University Malaysia. An example of a subject during a trial, is shown in Figure 1.

### Equipment and Apparatus

#### Motion Capture System

The motion capture (mo-cap) system used, was Qualisys, Sweden with a sampling frequency of 200 Hz. With this, two BERTEC force plates were used to detect the swing phase gait events—a TO and HS. The lower body segments of a subject were tracked (in mm) based on a 3D cartesian coordinate system, where the origin was placed within the calibrated area (force plates). Qualisys Track Manager (QTM) was the management and control software. It was used to record the mo-cap data of participants, synchronize and record data from the force plates and trigger the Biosignalsplux SEMG data logger (synchronization). This ensured the timescales of all individual systems matched. In addition, QTM was used to label and identify the mo-cap markers placed on the subjects.

#### Muscle Activity Retention Taping

MART was used to simulate elderly gait characteristics in young adults, focusing on the lower extremity muscles. MART was achieved by using two pairs of non-elastic blood flow restriction occlusion bands. The MART bands were placed on the shank and thigh segments of each foot. The bands on the shank were placed on the lower end of the *Gastrocnemius* and *Tibialis Anterior* to restrict the ankle's movements. While, the bands on the thigh were placed on the *Vastus Lateralis* and lower end of the *Rectus Femoris* to reinforce the reduction in joint motion and avoid compensation for the restriction on the ankle (5).

By restricting blood flow using MART, the increase in the transversal area of the muscle belly during contraction is reduced, leading to a decrease in the muscle length. This results in an increase in the stiffness of the muscle, a reduction in step length, walking velocity, ROM, and joint flexion. Thereby allowing the elderly gait characteristics (particularly a lower MTC) to be replicated in young adults (5).

The pressure range for safely restricting blood flow (due to MART), is between 180 and 200 mmHg. To ensure the pressure exerted, by each band is kept within this range—three force sensitive resistors (FSR-402) with an Arduino Uno, were used to maintain the restriction pressure at approximately 180 mmHg (10). This also ensures that each band exerts the same pressure, thus maintaining symmetry.

#### Surface Electromyography

Surface electromyography (SEMG) was used to measure the action potential (hyperpolarization and depolarization) of the three subject muscles—*Tibialis Anterior, Gastrocnemius Lateralis* and *Medialis*. A Biosignalsplux SEMG data logging kit was used to collect the readings through Opensignals software, which connected to the datalogger *via* Bluetooth. There were six channels/electrode pairs (one for each subject muscle) and a ground electrode, which was placed on a bone segment (collar bone). Opensignals was also used when finding the maximum SEMG reading for each muscle.

### Procedure

In the pretest phase—first the SEMG electrodes were placed on the six muscles with respect to the guidelines as stated by the SENIAM Project (Surface Electromyography for the Non-Invasive Assessment of Muscles) (11–13). A maximum voluntary contraction (MVC) for the three muscles on each foot were obtained. Participants were asked to tense their ankle with as much force as possible, to set a baseline for the maximum possible muscle activation. Next, reflective mo-cap markers were placed on the lower body segments of participants, as recommended by Visual 3D, C-Motion Inc. USA (14). One extra marker was placed on the Hallux of each foot, to measure MTC. The subject was then asked to stand in a stationary position with their arms crossed, and a single static trial was recorded. Next, six dynamic trials were recorded. Subjects were asked to walk (at their own pace) along a walkway spanning approximately 5 m. This ensured that they would reach their normal walking velocity and step length before reaching the force plates.

In the post-test phase—the MART bands were placed on the shank and thigh segments of each foot. The mo-cap markers were realigned and a static trial was obtained. Next, six more dynamic trials were obtained with muscle restriction. This concluded the experimental trials.

### Data Collection and Processing

Visual 3D and MATLAB were used in data processing to extract the MTC, SEMG readings and joint angles. SPSS Statistics V26.0, IBM was used for statistical analysis of the collected data.

On Visual 3D, the static trial was used to create a hybrid static model, which was then superimposed onto the dynamic trials to create the lower body bone segments. To normalize the ankle joint angles to zero (when the subject stood upright)—kinematic only, left and right feet were created within the static model.

For the dynamic trials, the marker trajectories were first interpolated with a fame gap of 10, to fill in missing data points (15). The trajectories were then filtered using a Butterworth low pass filter, with a cutoff frequency of 8 Hz. Visual 3D created the gait events (using the force plate readings), and the swing phase was identified between a TO and HS. The MTC, knee and ankle joint angles were then computed and exported into MATLAB for further analysis.

The TC during the swing phase splits into three sections. First it reaches a maximum, just after a TO, followed by a “trough” in the cycle—which corresponds to the MTC. Finally, the TC reaches a second maximum before the HS. MATLAB was used to find the MTC by considering the trajectory of the Hallux marker (in mm) at the trough. It can be noted that MTC occurs around mid-swing (50 to 60 percent of the swing phase) (16).

By noting down the times at which MTC occurred, the relevant joint angles were found, for that instant. The sign of the angle denotes its state:

- Extension is positive and flexion is negative (for the knee)
- Dorsiflexion is positive and plantar flexion is negative (for the ankle)
- Inversion is positive and eversion is negative
- Adduction is positive and abduction is negative

Next, the collected SEMG signal was processed. The signal was cropped to the required timeframe—between when the subject enters and leaves the calibrated area (~2 s), as taken from QTM. A Fast Fourier Transform of the signal was obtained and it was noted that the intensity of the peaks started to increase at around 30 Hz and reduced after 300 Hz. Hence, a 4th order Butterworth bandpass filter was applied with a highpass cut-off frequency of 30 Hz and a lowpass cut-off frequency of 300 Hz. This frequency range was identical to that, suggested for EMG signal processing in reference (17). The filter removed any high frequency noise to give a smoother envelope; and low frequency traces to remove movement artifacts and improve spectral resolution (17, 18). Next, the SEMG signal underwent full wave rectification. A root mean square (RMS) moving average envelope was computed (using the “movmean” function in MATLAB) to show the mean power of the signal. A sliding window size of 50 samples (corresponding to a 2.5 ms window) was used for a reasonable trade-off between roughness of the envelope and the area under it (19).

**Figure 2:**
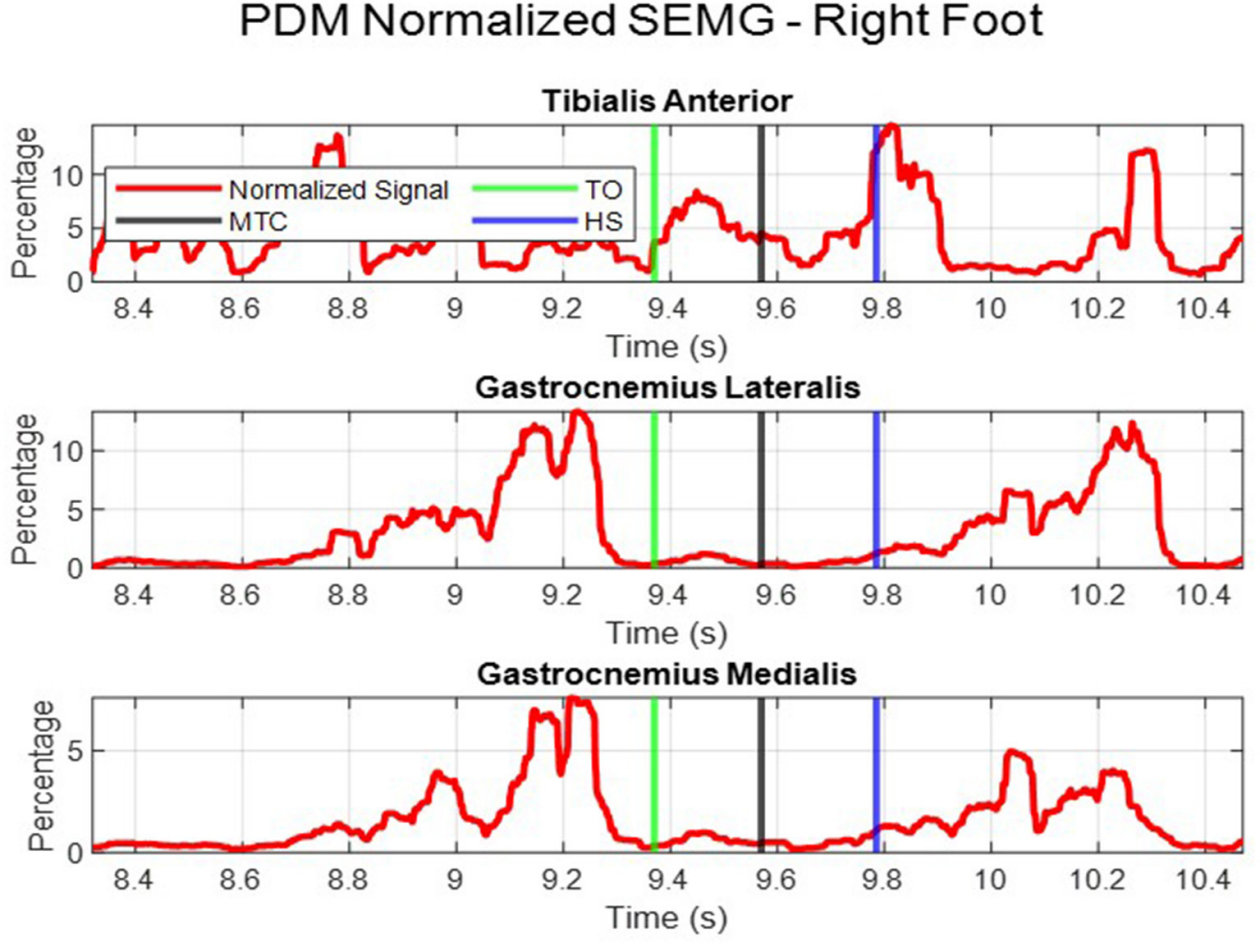
Normalized muscle activation using the PDM.

Finally, the SEMG signal was normalized using the peak dynamic method (PDM). The maximum muscle activation for each subject muscle was found by comparing the MVC and all other dynamic trials for that individual (20). The percentage of the RMS envelope to the PDM was then calculated and plotted. This is shown by the red curve in Figure 2. PDM gives a standardized signal to compare muscle activation across all subjects. For statistical analysis, the SEMG readings were obtained using two methods:

- The area under the normalized SEMG curve; during the swing phase (referred to as, “swing phase area” in this report).
- The individual muscle activations for each muscle at the time MTC occurs (referred to as, “at time MTC” in this report).

### Statistical Analysis

SPSS was used for analysis of the within subject's collected data. Initially, a one sample Kolmogorov-Smirnov test along with a Shapiro-Wilk test was used to test for normality. From the 12 data sets tested (Tables 1, 2), three were parametric. For these data sets the mean and standard deviations (SD) were considered while for the non-parametric data, the median and interquartile range (IQR) were used as measures of central tendency and variability, respectively (4).

**Table 1:** Results of kinematic parameters.

**Research variable**	**Normality test**	**Median/Mean**	**Pretest**	**Post test**
**Toe clearance (mm)**				
MTC (mm)	Non-parametric	Median (IQR)	24.10 (7.70)	18.95 (4.65)
MTC (normalized)	Non-parametric	Median (IQR)	0.014 (0.005)	0.011 (0.003)
**Joint angles at MTC (deg)**				
Knee flexion/extension	Non-parametric	Median (IQR)	−47.56 (6.58)	−41.32 (8.44)
Knee abduction/adduction	Non-parametric	Median (IQR)	4.79 (6.21)	−2.72 (10.41)
Ankle flexion/extension	Parametric	Mean (SD)	8.42 ± 4.37	6.81 ± 3.33
Ankle inversion/eversion	Non-parametric	Median (IQR)	13.58 (8.47)	17.04 (8.72)
Ankle abduction/adduction	Non-parametric	Median (IQR)	−3.61 (3.29)	−2.95 (3.41)

**Table 2:** Muscle activation (SEMG) results.

**Research variable**	**Normality test**	**Median/mean**	**Normalized muscle activation**	
			**Pretest**	**Post test**
*Tibialis Anterior* (Swing Phase Area)	Parametric	Mean (SD)	1617.54 ± 415.19	1726.55 ± 327.54
*Tibialis Anterior* (At MTC)	Parametric	Mean (SD)	3.64 ± 1.21	4.15 ± 1.55
*Gastrocnemius Lateralis* (Swing Phase Area)	Non-parametric	Median (IQR)	465.66 (302.25)	490.95 (194.21)
*Gastrocnemius Lateralis* (At MTC)	Non-parametric	Median (IQR)	0.93 (0.72)	1.00 (0.63)
*Gastrocnemius Medialis* (Swing Phase Area)	Non-parametric	Median (IQR)	403.87 (447.78)	419.92 (530.36)
*Gastrocnemius Medialis* (At MTC)	Non-parametric	Median (IQR)	0.72 (1.02)	0.97 (0.96)

Each subject performed six trials pre-MART and 6 trials with MART. The data values obtained from each trial, per subject, were considered as singular data points. Data (from all subjects) pre-MART were considered as pretest data and those with MART formed the post-test data. From the kinematic parameters, MTC was normalized with respect to the subject height (5); while the joint angles were normalized (to zero) when the subject stood in a static position. In addition, the SEMG data was normalized using the PDM, as discussed above.

Hypothesis testing was used to find if a significant difference existed in all variables; pre and post MART. For the parametric data sets, a paired samples *t*-test was used and for the non-parametric data, a Wilcoxon signed rank test. Each data set was compared within its pretest and post-test values to assess the simulated effects of aging (caused by MART). Following this, the intraclass correlation coefficient (ICC) was calculated for the post-test MTC (right and left feet), per subject. ICC can be used to measure the reliability (test re-test) and repeatability of the data collected and would indicate the level of similarity between collected MTC values per subject (21). The α value was 0.05 and the confidence interval was 95%.

## Results

### Minimum Toe Clearance

MTC was found to be non-parametric. This was confirmed by previously conducted studies, which showed that MTC was positively skewed and leptokurtic (4, 5). The overall median MTC with the IQR is shown in Table 1. During normal walking (for adults) the median MTC was between 10 and 20 mm (5). On the other hand, for the elderly, the median MTC was below 12.9 mm approximately (22). This study obtained a pretest median MTC of 24.10 mm and a post-test MTC of 18.95 mm.

A Wilcoxon signed rank test was applied to give a significance of *P* < α. Hence, a strong significant reduction in MTC after muscle restriction exists. This trend can also be seen by the decrease in the normalized median MTC from 0.014 before restriction to 0.011 after restriction.

### Joint Angles

As shown in Table 1, the joint angles for the knee and ankle were non-parametric, except ankle dorsiflexion/plantar flexion. Prior to muscle restriction, the overall trend at time MTC, placed the knee in flexion with a median angle of −47.56 degrees, and in adduction with a median angle of 4.79 degrees. After MART, there was a strong significant change in both knee flexion and adduction with *P* < α. The overall post-test trend saw the knee flexion decrease (become less negative) while the knee went into abduction.

The ankle was initially in dorsiflexion at time MTC, with a mean of 8.42 degrees. It was also in inversion with a median of 13.58 degrees and in abduction with a median of −3.61 degrees; pretest. The change in the angles from pretest to post-test is expressed by the significance, as such:

- Ankle dorsiflexion: *P* = 0.007, which is < α. Hence, a significant reduction in dorsiflexion was observed post-test.
- Ankle Inversion: *P* = 0.004, which is < α. Hence, a significant increase in inversion was observed post-test.
- Ankle abduction: *P* = 0.153, which is > α. Hence, no significant change in abduction was observed.

### Muscle Activation (Surface Electromyography)

Table 2 shows the mean and median SEMG readings for each subject muscle. For the *Tibialis Anterior*, there was a significant increase in muscle activation, for the swing phase area (*P* = 0.029) and at time MTC (*P* = 0.027). This is also reflected by the overall increase in the mean values for the *Tibialis Anterior*.

The *Gastrocnemius Lateralis* followed a similar trend to the *Tibialis Anterior*. There was a significant increase in muscle activation due to MART. For the swing phase area, the significance was *P* = 0.002 and for the value at time MTC the significance was *P* = 0.031. Both these values are < α and show a reasonable significance strength.

For the *Gastrocnemius Medialis*, there was a significant increase in swing phase area muscle activation (*P* = 0.005). However, for the muscle activation at time MTC, there was no significant change between the pretest and post-test data (*P* = 0.071). It should be noted, that on overall, the *Tibialis Anterior* had the highest muscle activation, both for the swing phase area and at time MTC. While the posterior muscles had a lower overall activation. The two subdivisions of the *Gastrocnemius* had similar muscle activation readings, as shown by their medians in Table 2.

## Discussion

The aim of this study was to investigate how the anterior and posterior muscles in the shank affect the level of MTC in elderly individuals. It was hypothesized, that the ankle is responsible for supporting the foot, during the swing phase. Therefore, the muscle responsible for ankle dorsiflexion—the *Tibialis Anterior—*should be the primary muscle that influences the level of MTC.

During SEMG signal analysis, the PDM gave a more accurate and reliable reading for the maximum activation of each muscle. When performing the MVC subjects may not contract their muscles with as much force as possible, hence there is no way to truly identify the maximum activation. Therefore, by comparing all muscle activation readings, across all trials, a better representation of maximum muscle activation can be found—justifying the use of PDM.

MART was used to simulate the gait characteristics of the elderly in young adults. From the results obtained, with MART, a significant decrease in MTC, knee flexion and ankle dorsiflexion were observed. The median MTC decreased to 18.95 mm yet, the literature showed that for elderly individuals MTC should be below 12.9 mm (22). The difference in these values, were due to an offset present in the TC readings—between the tip of the toe and the Hallux mo-cap marker.

The results are in-line with the expected trends from the study on MART (5). The significant decrease in MTC, along with significant reductions in knee flexion and ankle dorsiflexion mirror the main elderly gait characteristics; reproduced in the young adult participants. In addition, a section of the hypothesis can be confirmed as the ankle's dorsiflexion has reduced. This means, the ankle tends to bend down more, showing that it contributes to the reduction in TC.

With regards to MART—the ICC for post-test MTC from subjects 1–5 were 0.70, 0.75, 0.83, 0.80, and 0.83, respectively. This shows a high similarity between MTC values per subject. The strong ICC (close to 1) shows high reliability and reproducibility of the data collected. Hence, the results obtained were not due to distortion caused by the placement of the MART bands. This also shows that the subjects walked in the same way each time, with MART, further justifying the repeatability of this study.

The mean and median SEMG readings showed that the *Tibialis Anterior* had a stronger muscle activation compared to the *Gastrocnemius*. From this, it can be deduced, that the *Tibialis Anterior* is the prominent muscle active, both, throughout the swing phase and at the time MTC occurs, which follows the hypothesis. The relevance of this, is shown by the fact, that the *Tibialis Anterior* works throughout the swing phase to support the ankle (in dorsiflexion) and prevent it from dropping down. This is what maintains the level of TC; hence, it can be concluded that the *Tibialis Anterior* is the primary shank muscle that influences MTC.

On overall, for the swing phase area, the *Tibialis Anterior* and *Gastrocnemius* saw a significant increase in muscle activation. A similar trend was found for the muscle activation at the time of MTC. This was contrary to the hypothesis—which predicted that the overall muscle activation would decrease, with MART. This was due to the simulated aging, where elderly individuals would have weaker and stiffer muscles (lower muscle activation) and thus be unable to maintain a high level of MTC. However, for all three subject muscles, the activation was significantly greater with MART (considering swing phase area), yet MTC and the other relevant kinematic parameters saw a significant decrease.

As the subjects were young adults, with healthy and strong muscles (compared to an elderly individual), the three subject muscles would have worked harder to maintain the achieved level of TC. In addition, the results show that the muscles may have operated to counteract and overcome the external restriction placed on them, hence the higher activation readings. Regardless, as the muscles were active, and showed a significant change with MART, it can be concluded that the shank muscles do indeed play a vital role in influencing the MTC. Thus, for an elderly individual, weaker shank muscles would result in a greater ankle drop, during the swing phase and thereby, a lower TC.

The inversion of the ankle increased, along with the knee moving into abduction (pointing away from the midline of the body). This shows a more restricted walking pattern, where the subjects are tensing their ankle muscles with each step. Such characteristics would be expected due to the muscles being restricted and would further support the increase in muscle activity after restriction.

Furthermore, the muscle activation at the time MTC, also shows a significant increase after restriction, for the *Tibialis Anterior* and *Gastrocnemius Lateralis*. The *Gastrocnemius Medialis*, however, shows no significant change after restriction. The significance of the *Gastrocnemius Lateralis* (*P* = 0.031) is a weak significance, however the *Tibialis Anterior* has a comparatively higher significance of *P* = 0.027. This supports the conclusion that the *Tibialis Anterior* is the more dominant muscle in influencing TC.

A limitation of this study was a small sample size—hence, future work would include using a larger sample size to more strongly generalize the results, to a larger population. In addition, power analysis statistical tests would also be conducted in the future, to determine the required sample size and further justify the results obtained. Furthermore, the FSR-402 sensors used may have produced minute variations in the data due to varying deflections of the thigh muscles. In the future, an improved taping method should be investigated for enhanced symmetrical restriction when performing MART.

## Conclusion

The effects of aging on the anterior and posterior muscles in the shank, along with their impact on the level of MTC was investigated. Aging gait was simulated in young adults using MART. The results showed a significant decrease in the MTC, knee flexion and ankle dorsiflexion, which is expected due to aging. In addition, the knee adduction became abduction after restriction, while the ankle's inversion increased. No significant change in the ankle's abduction was observed.

The *Tibialis Anterior* was found to be the dominant muscle in the shank compared to the *Gastrocnemius* as it had a higher overall muscle activation. After restriction, the muscle activity increased (contrary to the hypothesis). This may be due to the subject's counteracting the external restriction–the muscles working harder to be able to at-most achieve the given MTC.

In conclusion, the anterior shank muscles can greatly influence MTC by controlling how far the ankle can drop during the swing phase. Weaker shank muscles, as seen in the elderly, would lead to a lower MTC and thus, a greater fall risk. The findings of this study can be used when designing devices and methods to monitor and improve TC in the elderly. It can be recommended that health care professionals, emphasize on strengthening the shank muscles, focusing on the *Tibialis Anterior*—to improve MTC and reduce fall risk.

## Data Availability Statement

The raw data supporting the conclusions of this article will be made available by the authors, without undue reservation.

## Ethics Statement

The studies involving human participants were reviewed and approved by Monash University Human Research Ethics Committee. The patients/participants provided their written informed consent to participate in this study.

## Author Contributions

AG conceived the study and the extent of its scope. CP performed the preliminary research and conducted the study. The methodology was finalized by CP, under the supervision of AG. The results for the study were obtained by CP, while the interpretation of the results and their respective conclusions were formulated by both CP and AG. The manuscript was written by CP under the supervision of AG. All authors read and approved the final manuscript.

## Conflict of Interest

The authors declare that the research was conducted in the absence of any commercial or financial relationships that could be construed as a potential conflict of interest.

## Abbreviations

ADC: Analog to digital converter
HS: Heel Strike
ICC: Intra-class Correlation
IQR: Inter-quartile range
MART: Muscle Activity Retention Taping
MTC: Minimum Toe Clearance
MVC: Maximum Voluntary Contraction
PDM: Peak Dynamic Method
QTM: Qualisys Track Manager
RMS: Root Mean Square
ROM: Range of Motion
SD: Standard Deviation
SEMG: Surface Electromyography
TC: Toe Clearance
TO: Toe Off.
